# Sickle Cell Disease: A Potential Cause of Immune Thrombocytopenia

**DOI:** 10.7759/cureus.76299

**Published:** 2024-12-24

**Authors:** Abdulwahab Ahmed A Alkhars, Mohammed A Alkadi, Hussain M Alwabari, Jagshak Mandong, Zahraa Y Almakinah

**Affiliations:** 1 Internal Medicine, King Fahad Hospital Hofuf, Hofuf, SAU

**Keywords:** adult sickle cell anemia, autoimmune disease, immune-mediated thrombocytopenia, immune thrombocytopenia, itp, platelet disorder, sickle cell complications, sickle cell crisis, sickle cell disease (scd), thrombocytopenia

## Abstract

Sickle cell anemia (SCA) is one of the known hemoglobinopathies that result in red blood cell (RBC) destruction, among other complications. There are factors that make SCA an environment for autoimmune disease (AID). They include chronic inflammation, immune-mediated processes involved in SCA complications, and susceptibility to infections. Furthermore, immune thrombocytopenia (ITP) is a complex disorder characterized by immune imbalance that results in platelet destruction.

We report a case of a 20-year-old patient with SCA presenting with a vaso-occlusive crisis (VOC) and unexplained thrombocytopenia. After ruling out secondary causes of the latter condition, the patient was challenged with steroid therapy. Interestingly, he showed a dramatic response in platelet count.

This report emphasizes the potential role of SCA in the development of ITP and encourages further studies to explore the underlying immunological mechanisms.

## Introduction

Sickle cell anemia (SCA) is characterized by chronic hemolysis, inflammation, and the presence of poorly deformable red blood cells (RBCs). Any condition that may further impair blood rheology, increase hemolysis, oxidative stress, and/or inflammation, may aggravate the disease. Autoimmune disease (AID) is a pathological condition caused by the adaptive immune system, where an autoimmune response results in tissue damage or organ dysfunction. The clinical expression of AIDs usually relates to inflammation-related damage to the target organ, with subsequent dysfunction [1].

SCA can manifest with thrombocytopenia through different mechanisms, such as splenic sequestration and aplastic anemia [2,3]. Identification of thrombocytopenia etiology can influence the management, as in the case of hydroxyurea (a disease-modifying therapy for sickle cell disease)-induced thrombocytopenia, and in immune thrombocytopenia (ITP) [4,5].

This case report explores a potential link between SCA and ITP. ITP itself is a complex disorder, with multiple factors and various cell types contributing to the immune imbalance resulting in platelet destruction. The primary mechanisms involved include anti-platelet antibody-mediated platelet phagocytosis and/or T-cell-mediated platelet destruction [5].

## Case presentation

History

We present a 20-year-old Saudi male with a known history of SCA, who was referred to the Hematology Department at King Fahad Hospital, Hofuf, Saudi Arabia, from a private hospital for evaluation of unexplained thrombocytopenia. The patient initially presented to his primary care provider with complaints of chest and lower limb pain, consistent with a vaso-occlusive crisis (VOC).

He had no history of bleeding tendencies, including epistaxis, gum bleeding, easy bruising, or spontaneous ecchymoses. There were also no symptoms suggestive of gastrointestinal, genitourinary, or central nervous system bleeding. While his presentation included pain suggestive of vaso-occlusion, there were no additional signs or symptoms to indicate thrombosis, such as swelling, redness, or warmth in the affected limbs. The patient denied recent trauma, infection, or any constitutional symptoms, such as fever, night sweats, or unexplained weight loss. He reported no gastrointestinal symptoms, like nausea, vomiting, or diarrhea, and had no history of peptic ulcer disease. There were no clinical indications of focal infection, recurrent infections, or systemic autoimmune disorders.

The patient is a smoker with a one- to two-year history of use, but he denied any use of herbal supplements. His routine medications include folic acid and occasional mild analgesia (acetaminophen). He is not on hydroxyurea and has had only infrequent hospitalizations, with no prior history of blood transfusions or acute chest syndrome. During his most recent hospitalization at the private facility, he was treated for VOC with analgesics and adequate hydration. During his stay, a significant decrease in platelet count was noted, prompting a referral to our facility for further evaluation.

Clinical examination

On examination, the patient was vitally stable. He was alert, conscious, and fully oriented to time, place, and person. There were no signs of skin lesions, such as ecchymosis, purpura, or petechial rashes. No lymphadenopathy or organ enlargement was noted. There were no clinical signs suggestive of deep vein thrombosis (DVT) or AIDs, such as joint swelling, malar rashes, or other systemic manifestations. Additionally, no neurological deficits, such as weakness, sensory loss, or abnormal reflexes, were observed. His cardiopulmonary examination was within normal limits.

Investigations

The patient’s initial laboratory investigations and workup of secondary causes of ITP are shown in Tables 1-2. The platelet count was 19 x 10^9. However, hemoglobin (Hb) and white blood count were in the normal range, making spleen disorder or bone marrow disease unlikely. Antinuclear antibody (ANA) is positive with a titer of 1/160. This positivity is in low titer and is associated with negative results for other immunological markers. Notably, the patient has no clinical manifestations fulfilling diagnostic criteria for any AIDs, like systemic lupus erythematosus. Hb electrophoresis is consistent with SCA. Also, it showed persistent fetal Hb. The blood film showed true thrombocytopenia and no abnormal cells. Possible secondary causes of ITP were negative. These include hepatitis B and C, human immunodeficiency virus (HIV), B12 deficiency, folate deficiency, thyroid disease, and *Helicobacter pylori* infection. Elevated lactate dehydrogenase could indicate a hemolysis process, which is compensated, as the Hb level is normal.

**Table 1:** Initial labortory investigations and work up of secondary causes of immune thrombocytopenia ANA, Antinuclear antibody; SSA/SSB, Sjögren syndrome antibody A/Sjögren syndrome antibody B; SM, Smith antigen; RNP, Ribonucleoprotein; dsDNA, Double-stranded DNA

Test		Result	Reference range
Complete blood count	Hemoglobin	13.4 g/dL	Male 13 - 16.5; female 12 - 16
	White blood cells	6.0 x 10^9/L	4.5 x 10^9 - 1000 x 10^9
	Platelet	19 x 10^9/L	15 x 10^9 - 450 x 10^9
Renal function test	Blood urea nitrogen	3.3 mmol/L	3.2 - 7.1
	Creatinine	61 µmol/L	52 - 120
Hemoglobin electrophoresis	Hemoglobin S	62.80%	0%
	Hemoglobin A	0%	96.5% - 98%
	Hemoglobin A2	2.1%	1.5% - 3.5%
	Hemoglobin F	33.2%	0% - 1%
Immunology profile	ANA	Positive (1/160)	Negative
	Anti-SSB	Negative	Negative
	Anti-SSA	Negative	Negative
	Anti-SM	Negative	Negative
	Anti-RNP	Negative	Negative
	Anti-dsDNA	Negative	Negative
Virology profile	Hepatitis B surface antigen	Negative	Negative
	Hepatitis C virus antibody	Negative	Negative
	Human immunodeficiency virus antigen/antibodies	Negative	Negative

**Table 2:** Blood film and further work up of secondary causes of immune thrombocytopenia

Test	Result	Reference range
Blood film	Thrombocytopenia, no clumps, and occasional giant platelets	-
*Helicobacter pylori* antigen in stool	Negative	Negative
Thyroid-stimulating hormone	1.088 µIU/mL	0.465 - 4.68
Vitamin B12	290	211 - 911
Folate	>20 ng/mL	>4 ng/mL
Lactate dehydrogenase	1527	120 - 246

Management

The patient's progressive low platelets, as well as the exclusion of other potential causes, led to the diagnosis of ITP based on the patient's illness course and laboratory findings. We immediately initiated the management plan, which involved administering dexamethasone 40 mg IV once daily for four days.

Outcome

The patient showed a dramatic response in platelet count after steroid therapy. A drop in platelet count was noted during a five-month follow-up. This is expected in chronic ITP. Importantly, as the platelet count is above 20 and there is no bleeding, this is consistent with remission, with no indication for second-line treatment. The platelet count trend is summarized in Table 3.

**Table 3:** Platelet count before and after therapy

Platelet count			
Initial count	After steroid challenge	After five-month follow-up	Normal range
29 x 10^9	427 x 10^9	73 x 10^9	15 x 10^9 - 450 x 10^9

## Discussion

ITP is an acquired autoimmune disorder characterized by a low platelet count, resulting from platelet destruction and impaired platelet production. It has an incidence of 2 to 5 per 100,000 and can present as either an isolated primary condition or in association with other underlying diseases. As a heterogeneous disorder with variable clinical manifestations, ITP is often considered a diagnosis of exclusion, after ruling out other causes of thrombocytopenia [6].

A key aspect of ITP is that it can also be secondary to a wide range of underlying conditions, a concept often referred to as secondary ITP. Secondary ITP encompasses all forms of immune-mediated thrombocytopenia arising from an associated disease or drug exposure. This form of ITP is linked to various autoimmune disorders, including systemic lupus erythematosus and antiphospholipid syndrome, as well as immunodeficiencies such as IgA deficiency and common variable immunodeficiency. It can also occur in the context of lymphoproliferative disorders, like chronic lymphocytic leukemia and lymphoma, and in infections with viruses such as HIV, *H. pylori*, cytomegalovirus (CMV), and HCV. Additionally, certain medications, including heparin and quinidine, can trigger secondary ITP [7].

While SCA has not been directly established as a cause of ITP, it is associated with various immune dysregulations that could potentially increase the risk of autoimmune complications. A total of 338 patients with SCA were involved in a retrospective study assessing the prevalence of AIDs in SCA patients. Of these, 36 (10.7%) had been diagnosed with at least one AID. Among them, 14 different AIDs were diagnosed in these 36 patients; the most frequent (>1%) diagnosed AIDs were sudden deafness (1.8%), hyperthyroidism and hypothyroidism (3%), and sarcoidosis (1.2%) [8].

In this report, we present a potentially novel case of ITP in a patient with SCA, diagnosed by the exclusion of secondary causes and demonstrating a remarkable response to dexamethasone. This case raises the intriguing possibility that SCA itself may act as a secondary cause of ITP due to its associated immune dysregulation. The well-established association between SCA and autoimmune disorders further supports this hypothesis, suggesting that SCA may predispose individuals to AIDs, including ITP.

Notably, our case was safely treated with dexamethasone, despite corticosteroids being used with caution in patients with SCA due to the increased risk of rebound vaso-occlusive pain events [9].

From a mechanistic point of view, SCA has the ability to create a favorable microenvironment for autoimmunity to develop. Chronic inflammation and endothelial cell activation result in the release of cytokines, such as tumor necrosis factor-α, interleukin (IL)-6, and IL-1, with subsequent activation of cells of the immune system. Increased susceptibility to infections in SCA probably has a similar effect, contributing to long-term exposure of the immune system to a variety of antigens. Furthermore, there is an increased presence of microparticles in the circulation, originating from RBCs, platelets, monocytes, and endothelial cells, both in a steady state and during acute crises. These microparticles are released due to cell damage or apoptosis and exhibit altered membrane properties, including the exposure of phosphatidylserine, which attracts phagocytic cells [10].

Interestingly, RBC-derived microparticles are prothrombotic and can activate the complement system. Additionally, microparticles from other cell types contain pro-inflammatory molecules, such as DNA, RNA, and histones, which can serve as autoantigens. Overall, these microparticles may circulate in the bloodstream, interact with specific tissues, and promote immune activation, including the formation of immune complexes [10].

Patients with SCA also exhibit elevated titers of autoantibodies, prompting the question of whether SCA predisposes individuals to a higher prevalence of clinical AIDs. A 2021 study by the American Society of Hematology reported that AIDs are more common in SCA patients, with a prevalence of 10.7%, higher than that in the general population. This finding was revealed in retrospective studies from Amsterdam and the UK, which found increased rates of autoimmune conditions, including systemic lupus erythematosus and connective tissue disease, in SCA patients [11]. These observations suggest that SCA may play a role in the development of AIDs, including ITP.

Our case report contributes to this growing body of evidence, supporting the potential link between SCA and ITP, and underscoring the need for further research in this area. As the immune dysregulation in SCA becomes increasingly recognized, it is reasonable to consider SCA as a potential secondary cause of ITP.

## Conclusions

This report emphasizes the potential role of SCA in the development of ITP and encourages further studies to explore the underlying immunological mechanisms. While the association between SCA and AIDs is well-documented, the specific link between SCA and ITP remains an area for exploration. More case reports, along with prospective studies, are needed to define the relationship between SCA and autoimmune thrombocytopenia, which could ultimately improve the management of SCA patients who present with autoimmune complications.
